# Supplementary figures and images for: Morphological evolution and diversity of pectoral fin skeletons in teleosts
Source: Zoological Lett. 2022 Nov 26;8:13. doi: 10.1186/s40851-022-00198-y (PMC9701400; doi:10.1186/s40851-022-00198-y)

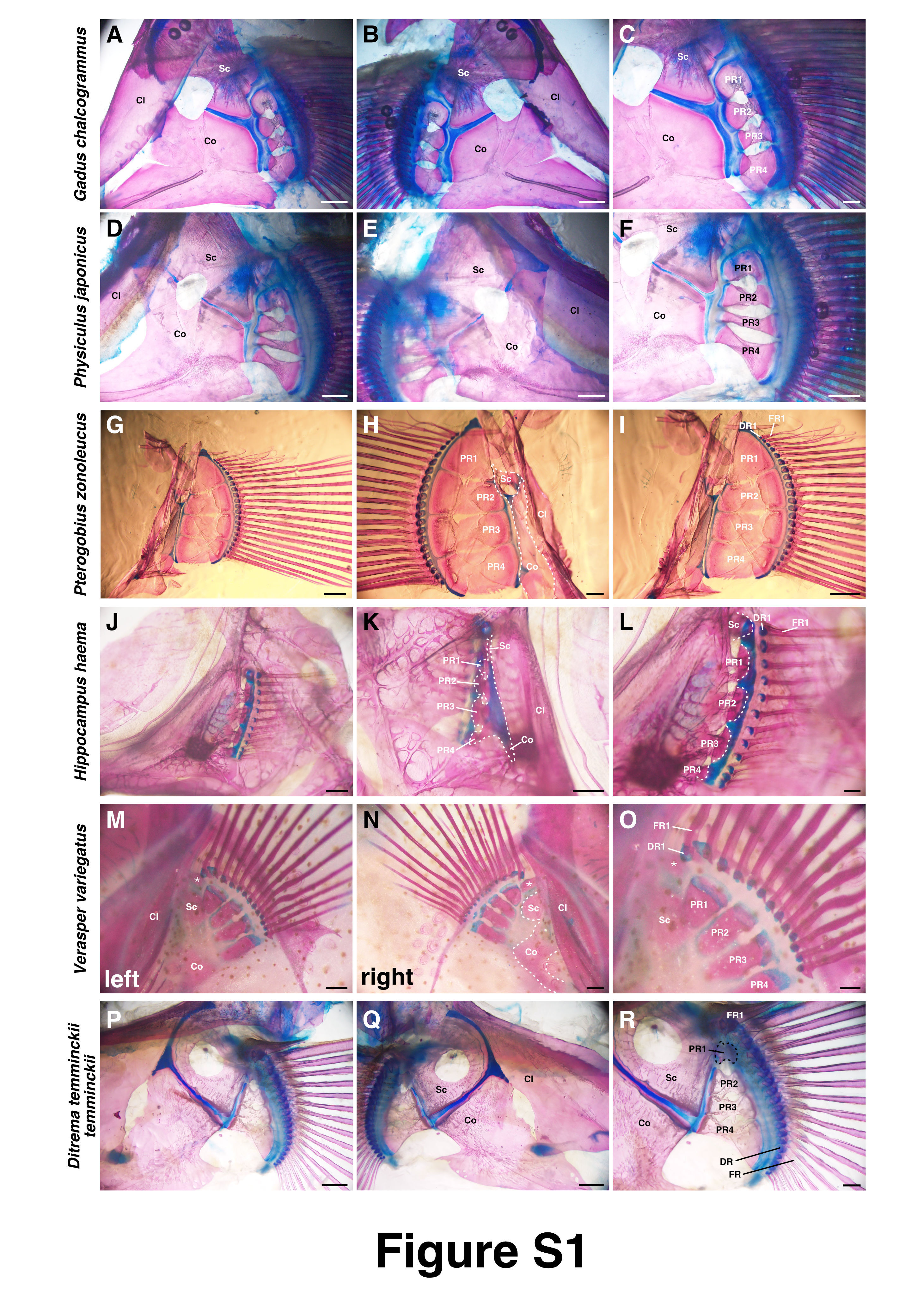

Supplement: Supplementary file 1 — Additional file 1: Fig. S1. Pectoral fin skeletons of Paracanthopterygii (A–F) and Acanthopterygii excluding Eupercaria (G–R). (A–C) Pectoral fin skeleton of Gadus chalcogrammus (32 cm TL) observed from the lateral view (A, C) and medial view (B). (D–F) Pectoral fin skeleton of Physiculus japonicus (24 cm TL) observed from the lateral view (D, F) and medial view (F). (G–I) Pectoral fin skeleton of Pterogobius zonoleucus (5.2 cm TL) observed from the lateral view (G, I) and medial view (H). (J–L) Pectoral fin skeleton of Hippocampus haema (4.0 cm measured between the top of the head and the farthest point on the curved tail from the head) observed from the lateral view (J, L) and medial view (K). (M–O) Pectoral fin skeletons of Verasper variegatus (2.5 cm TL) on the left side (M, O) and the right side (N); white asterisk indicates propterygium-like bone. (P–R) Pectoral fin skeleton of Ditrema temminckii temminckii (16 cm TL) observed from the lateral view (P, R) and mesial view (Q). Cl, cleithrum; Co, coracoid; DR, distal radial; FR, fin ray; PR, proximal radial; Sc, scapula. Scale bars: 2 mm (A, B, D–F, Q); 1 mm (C, G, I, P, R); 500 µm (H, J, K); 200 µm (L, M, N); 100 µm (O). [file 40851_2022_198_MOESM1_ESM.jpg]

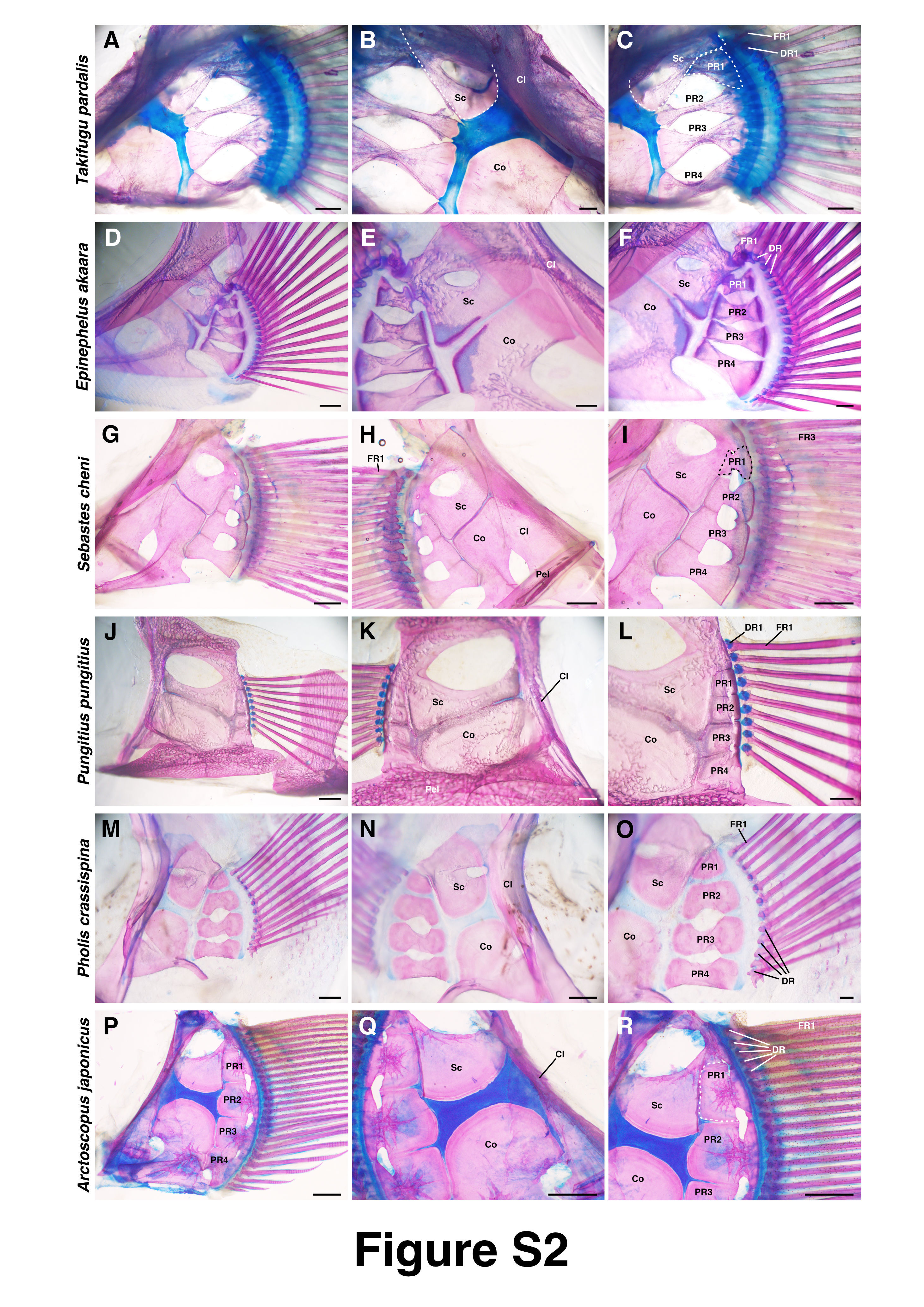

Supplement: Supplementary file 2 — Additional file 2: Fig. S2. Pectoral fin skeletons in Eupercaria, a major group of Acanthopterygii. (A–C) Pectoral fin skeleton of Takifugu pardalis (18 cm TL) observed from the lateral view (A, C) and medial view (B). (D–F) Pectoral fin skeleton of Epinephelus akaara (6.5 cm TL) observed from the lateral view (D, F) and medial view (E). (G–I) Pectoral fin skeleton of Sebastes cheni (22 cm TL) observed from the lateral view (G, I) and medial view (H). (J–L) Pectoral fin skeleton of Pungitius pungitius (5.5 cm TL) observed from the lateral view (J, L) and medial view (H). (M–O) Pectoral fin skeleton of Pholis crassispina (11 cm TL) observed from the lateral view (M, O) and medial view (N). (P–R) Pectoral fin skeleton of Arctoscopus japonicus (22 cm TL) observed from the lateral view (P, R) and medial view (Q). Cl, cleithrum; Co, coracoid; DR, distal radial; FR, fin ray; Pel, pelvic girdle; PR, proximal radial; Sc, scapula. Scale bars: 4 mm (G–I, P–R); 2 mm (A, C); 1 mm (B, D, J); 500 µm (E, F, K–N); 200 µm (O). [file 40851_2022_198_MOESM2_ESM.jpg]

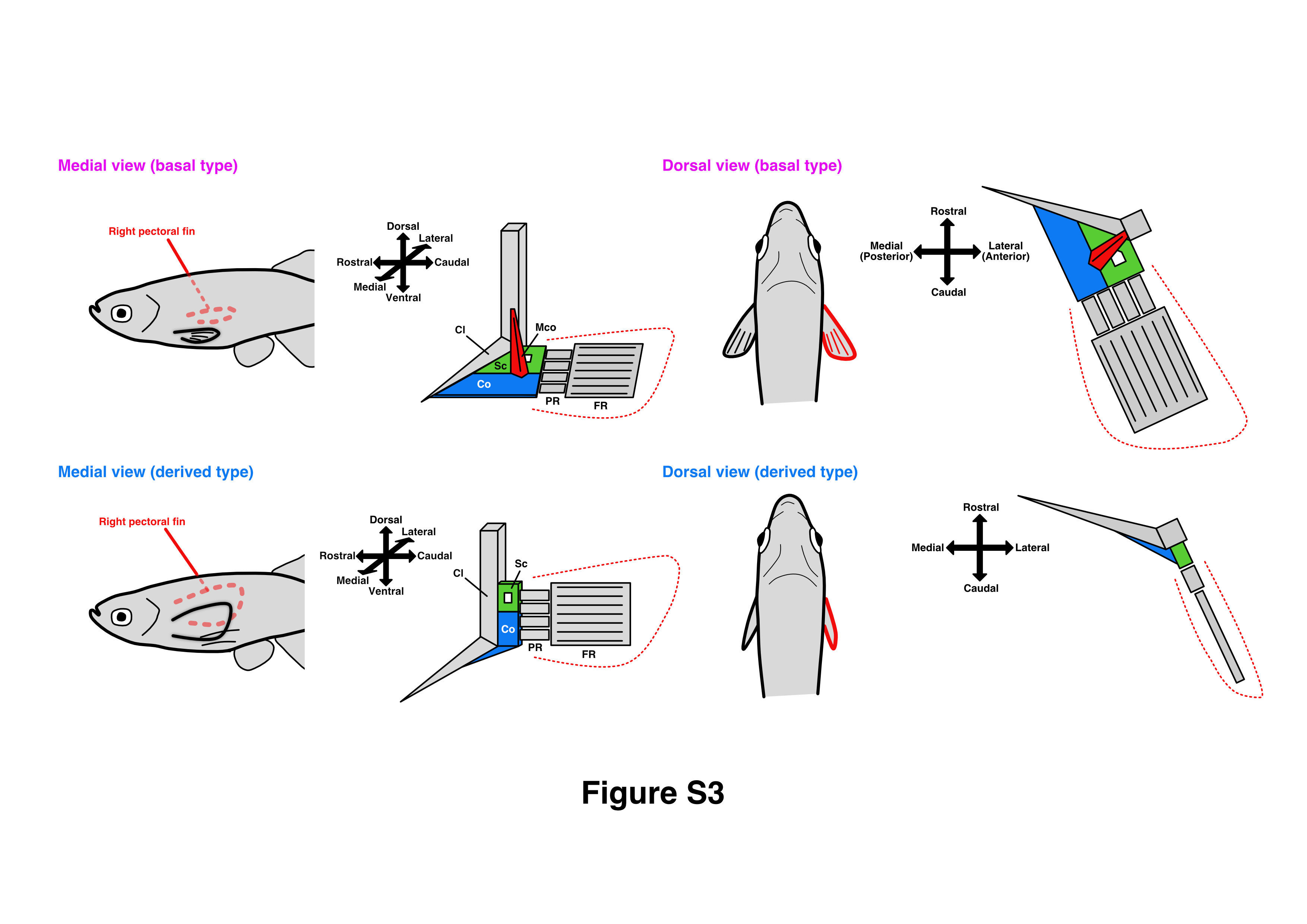

Supplement: Supplementary file 3 — Additional file 3: Fig. S3. Schematic diagrams of morphological comparison of pectoral fin skeletons focused on differences of girdle components between basal (mainly Osteoglossomorpha, Otomorpha, Protacanthopterygii and Stomiati) and derived (mainly Paracanthopterygii and Acanthopterygii) pectoral fin skeletons. Cl, cleithrum; Co, coracoid; FR, fin ray; Mco, mesocoracoid; Pel, pelvic girdle; PR, proximal radial; Sc, scapula. [file 40851_2022_198_MOESM3_ESM.jpg]
